# Bis(monoacylglycero)phosphate lipids in the retinal pigment epithelium implicate lysosomal/endosomal dysfunction in a model of Stargardt disease and human retinas

**DOI:** 10.1038/s41598-017-17402-1

**Published:** 2017-12-11

**Authors:** David M. G. Anderson, Zsolt Ablonczy, Yiannis Koutalos, Anne M. Hanneken, Jeffrey M. Spraggins, M. Wade Calcutt, Rosalie K. Crouch, Richard M. Caprioli, Kevin L. Schey

**Affiliations:** 1 Mass Spectrometry Research Center and Department of Biochemistry, Vanderbilt University School of Medicine, Nashville, TN USA; 2 Department of Ophthalmology, Storm Eye Institute, Medical University of South Carolina, Charleston, SC USA; 3 Preclinical Department, Ora Inc, Andover, MA USA; 4 Department of Molecular and Experimental Medicine, The Scripps Research Institute, La Jolla, CA, USA; 5 Department of Biochemistry, Vanderbilt University, Nashville, TN USA; 6 Department of Chemistry, Nashville, TN USA; 7 Department of Pharmacology and Medicine, Vanderbilt University, Nashville, TN USA

## Abstract

Stargardt disease is a juvenile onset retinal degeneration, associated with elevated levels of lipofuscin and its bis-retinoid components, such as *N*-retinylidene-*N*-retinylethanolamine (A2E). However, the pathogenesis of Stargardt is still poorly understood and targeted treatments are not available. Utilizing high spatial and high mass resolution matrix assisted laser desorption ionization (MALDI) imaging mass spectrometry (IMS), we determined alterations of lipid profiles specifically localized to the retinal pigment epithelium (RPE) in *Abca4* ^*−/−*^ Stargardt model mice compared to their relevant background strain. Extensive analysis by LC-MS/MS in both positive and negative ion mode was required to accurately confirm the identity of one highly expressed lipid class, bis(monoacylgylercoro)phosphate (BMP) lipids, and to distinguish them from isobaric species. The same BMP lipids were also detected in the RPE of healthy human retina. BMP lipids have been previously associated with the endosomal/lysosomal storage diseases Niemann-Pick and neuronal ceroid lipofuscinosis and have been reported to regulate cholesterol levels in endosomes. These results suggest that perturbations in lipid metabolism associated with late endosomal/lysosomal dysfunction may play a role in the pathogenesis of Stargardt disease and is evidenced in human retinas.

## Introduction

Retinal degeneration is a complex multifactorial process ultimately resulting in death of the light sensitive photoreceptors present in the outer retina^1^. Stargardt disease is a juvenile form of autosomal recessive macular degeneration most commonly associated with defects in the ABCA4 gene^2–4^. This gene codes for the ABCA4 transporter, an ATP cassette binding protein present in the outer segments of the photoreceptor cells in the outer retina^4^. ABCA4 facilitates the translocation of phosphatidylethanolamine (PE) and Schiff bases with retinaldehyde from the intradiskal to the cytosolic side of the disk membrane.

With the processes of photoreceptor outer segment disk shedding and phagocytosis, the outer segment material ultimately enters the lysosomal compartment of the retinal pigment epithelium

(RPE)^5–7^. As buildup of proteins and damaged organelles occurs in the RPE, lipofuscin granules increase in number with age^8^, resulting in an increase in total lipofuscin fluorescence. Due to the excessive accumulation of lipofuscin observed when the ABCA4 gene is defective (mutated or missing)^9^, it is thought lipofuscin contributes to the pathogenesis of the disease. The molecular composition of lipofuscin has not been fully characterized although N-retinylidene-N-retinylethanolamine (A2E), a major fluorophore found in lipofuscin, has been extensively studied^6,10–17^. This cationic amphiphilic derivative of *N*-retinylidene phosphatidylethanolamine^18^, accumulates rapidly in both Stargardt patients as well as in the *Abca4* ^*−/−*^ knockout model. A2E has previously been shown *in vitro* to inhibit phagolysosomal degradation of photoreceptor outer segments resulting in perturbations in cholesterol metabolism and delayed lipid degradation *in vitro* ^19–21^. Therefore, the accumulation of A2E in Stargardt is thought to play a leading role in the pathogenesis of the disease. A similar process has also been proposed to play a role in age-related macular degeneration (AMD)^22–26^.

Matrix assisted laser desorption ionization imaging mass spectrometry (MALDI IMS) is a powerful analytical tool that has the ability to map the spatial distribution of lipids, proteins, and metabolites to specific regions within tissue with high spatial accuracy and chemical specificity^27–38^. Previous reports using MALDI IMS on retinal tissue include analysis from a variety of species including salamander^39^, mouse^12,40^, pig^36^, monkey^41,42^ and human tissues^35,39–42^. Advancements in instrumentation and sample preparation techniques have resulted in retinal images with improved image quality providing high spatial accuracy and allowing molecular signatures to be mapped to specific cell layers, including the single cell layer of the RPE^11,12,23–25,35,43,44^. Moreover, the use of high mass resolution and high mass accuracy measurements afforded by Fourier transform ion cyclotron resonance (FTICR) instruments allows unprecedented depth of analysis in the IMS experiments^33,38,45^.

Here we present a multimodal approach bringing together MS-based imaging technologies and LC-MS/MS approaches to identify molecular species which may be involved in retinal degradation in both *Abca4* ^*−/−*^ knockout mouse tissue and human retinal tissue. The RPE and photoreceptor layers were able to be spatially and molecularly differentiated using a combination of high chemical specificity and high spatial resolution provided by MALDI FTICR IMS experiments. Subsequent targeted liquid chromatography tandem mass spectrometry (LC-MS/MS) enabled identification of multiple bis(monoacylglycero)phosphate lipids species (BMP), a structurally unique family of lipids that have been observed in a number of endosomal/lysosomal storage diseases^46–48^. These results provide evidence of lipid disturbances and potential endosomal/lysosomal defects in the RPE spatially resolved across the tissues in an animal model of Stargardt disease. The presence of the same lipids in human donor tissue is consistent with the concept that lipid disturbances and potential endosomal/lysosomal defects in the RPE are likely to play a role in the pathogenesis of Stargardt disease and possibly other retinal disorders.

## Results

### Accumulation of specific lipid species in the Stargardt model RPE

**Figure 1:**
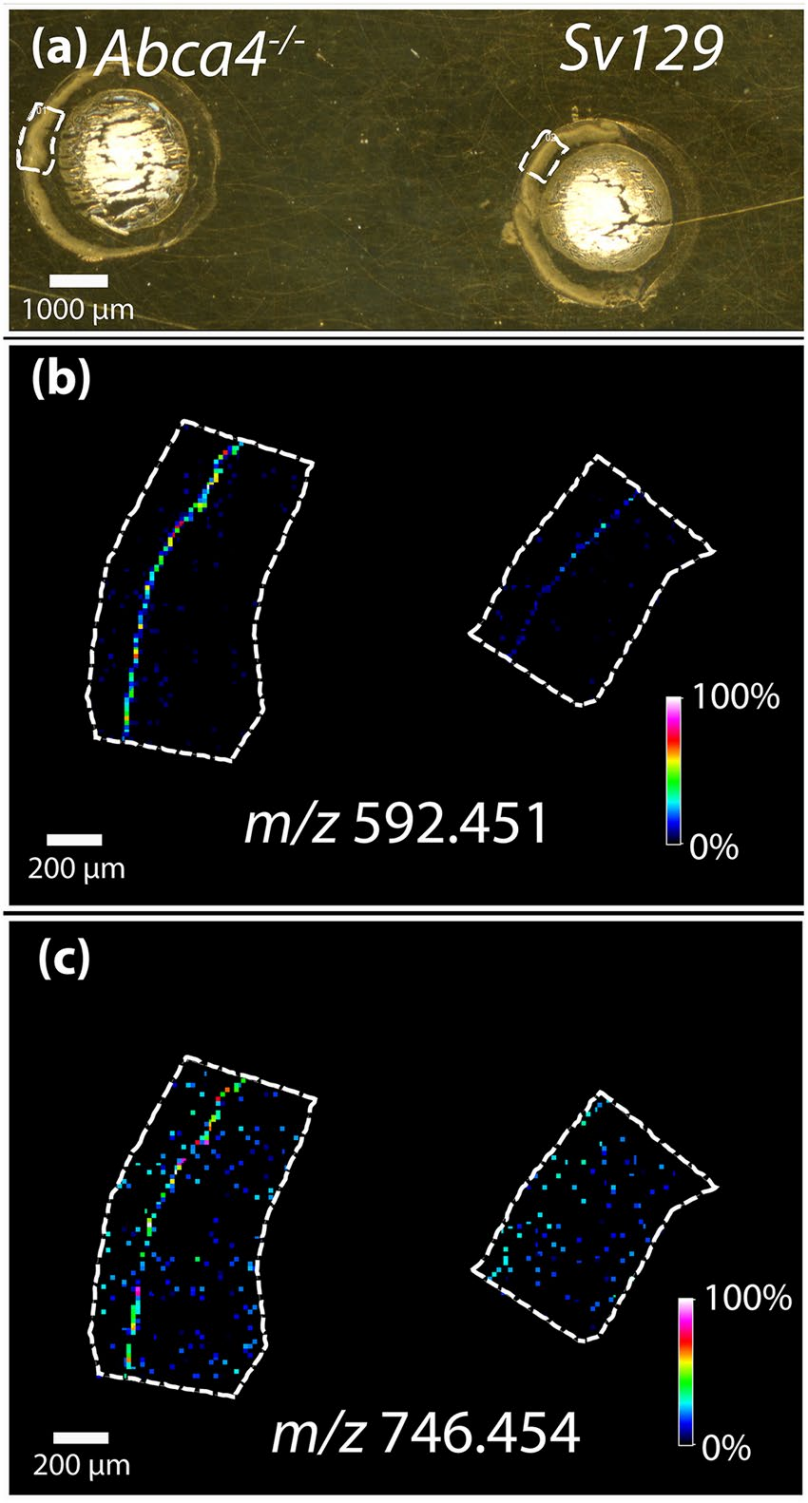
MALDI FTICR IMS analysis of *Abca4* ^*−/−*^ and *Sv129* ^*−/−*^ mouse retina tissues in positive ion mode. (**a**) Optical image of tissue, dotted line indicating region where data were acquired. MALDI IMS images displaying the relative abundance of (**b**) *m/z* 592.451 and *m/z* 746.454 (**c**).

Figure 1a displays optical images of tissue from *Abca4* ^*−/−*^ and *Sv129* mouse retina with the regions of interest selected for IMS analysis indicated by the dotted line. Selected positive ion MALDI IMS images (Fig. 1b) show high relative abundance of the A2E signal, identified by accurate mass measurement of *m/z* 592.451, in the *Abca4* ^*−/−*^ mouse model tissue. Note that A2E and A2GPE were previously identified using accurate mass measurements and MS/MS fragmentation patterns from *Abca4* ^−/−^ tissue extracts^12^. An MS/MS spectrum of an A2E standard is shown in Supplementary Figure 1. Low intensity A2E signal is indicated by the line of blue pixels observed in the RPE region of the control tissue and is attributed to A2E accumulation with normal aging. A related metabolite, A2GPE^13^, was also observed in the RPE layer of the *Abca4* ^*−/−*^ mouse model at *m/z* 746.454 (Fig. 1c). Low relative abundance was observed in the RPE of the *Abca4* ^*−/−*^ mouse with no signal detected in the *Sv129* tissue. These findings were replicated in triplicate from adjacent sections from the same animal using an FTICR mass analyzer and in two separate animals for each strain, i.e. two additional *Abca4* ^*−/−*^ animals and two additional *Sv129* animals, using a time of flight (TOF) mass analyzer, shown in Supplementary Fig. 2.

**Figure 2:**
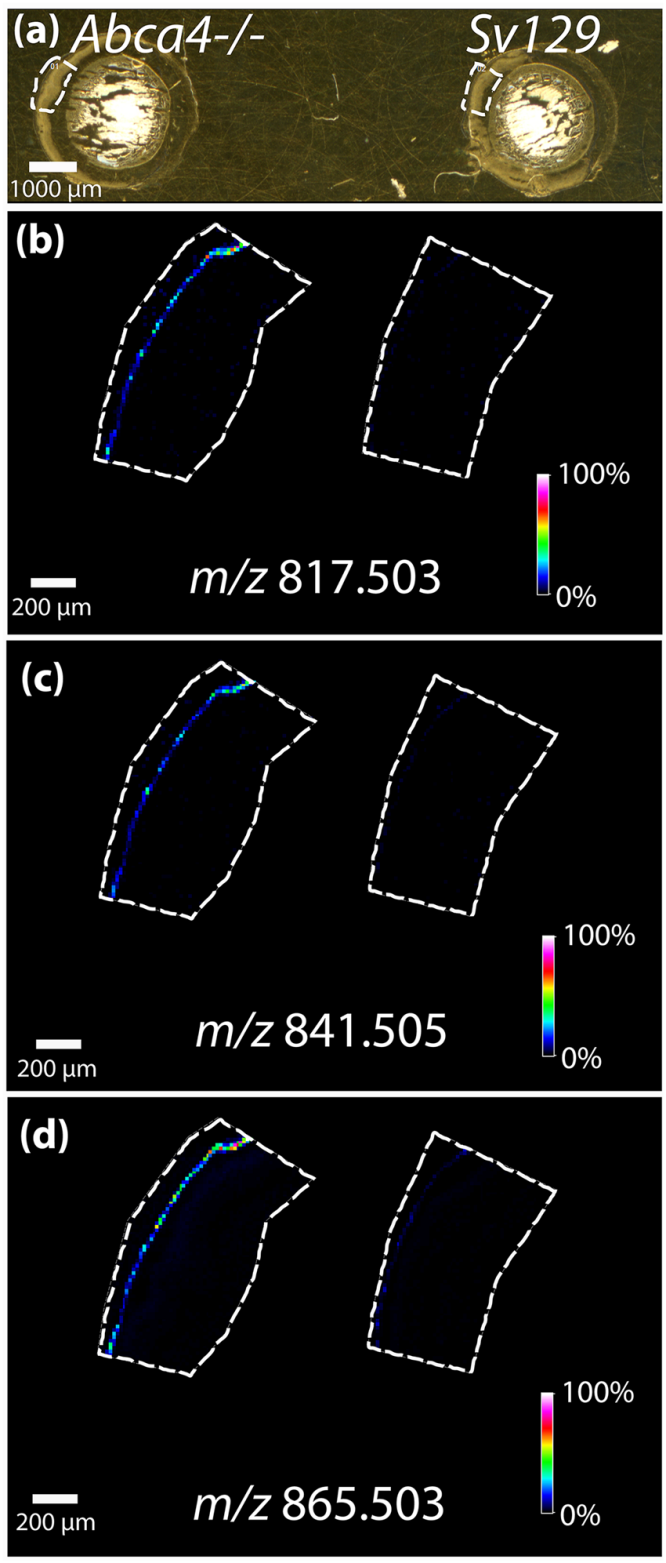
MALDI FTICR IMS analysis of mouse tissues *Abca4* ^*−/−*^ and *Sv129* in negative ion mode. (**a**) Optical image of tissue, dotted line indicating region where data were acquired. MALDI IMS images displaying the relative abundance of (**b**) *m/z* 817.503, (**c**) *m/z* 841.505, and (**d**) *m/z* 865.503.

Negative ion mode FTICR IMS data highlighting three signals observed in high abundance in the RPE region of the *Abca4* ^*−/−*^ tissue are shown in Fig. 2. Figure 2a represents an optical image of the retinal tissue with the regions of interest selected for IMS analysis indicated by the dotted line. The ions at *m/z* 817.503 (b), *m/z* 841.505 (c) and *m/z* 865.503 (d) were observed to localize to the RPE layer (Fig. 2b) and have not been observed in previous IMS experiments of ocular tissues. Only very low, almost undetectable signals were observed for these ions in the *Sv129* retina. Replicates of these data can be seen in Supplementary Figs 3 and 4. Within our detection limits no other major differences were observed between the RPE/photoreceptor region in these tissues.

**Figure 3:**
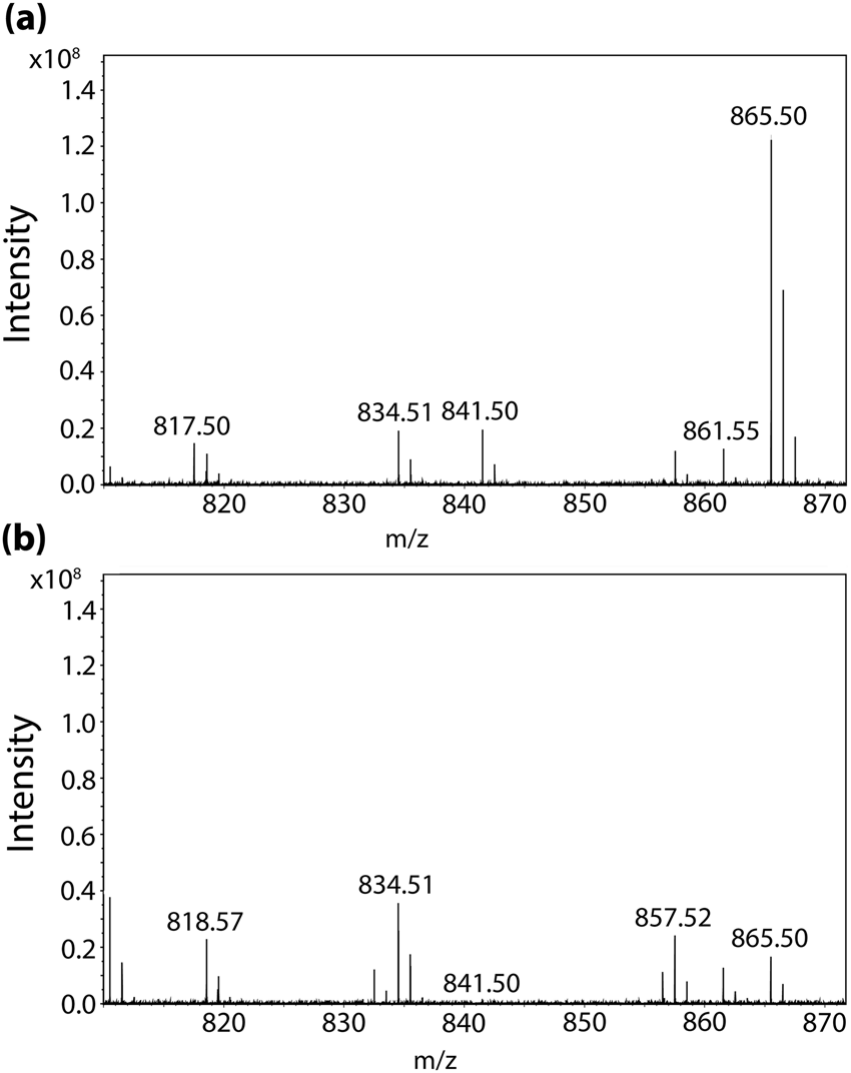
MALDI mass spectra taken from a single pixel in the RPE region of tissue from (**a**) *Abca4* ^*−/−*^ and (**b**) *Sv129* mouse tissue.

Mass spectra in the mass range of m/z 810–870 were exported from a single pixel of high intensity observed in the imaging data from the RPE region of each of the tissues analyzed in negative ion mode and are shown in Fig. 3 to show the molecular profiles generated from the tissue. The most intense peaks are labeled with their accurate mass. The spectra show that three peak distinctive peaks observed in Fig. 2, m/z 817.50, 841.50 and 865.50 are more highly abundant in the *Abca4* ^*−/−*^ mouse model compared to the control. A database search (lipidmaps.org) for *m/z* 865.503 (+/− 0.01 Da) suggests PG(22:6/22:6) as the lipid corresponding to the mass with a 0.6 ppm error. This identification was previously reported from MALDI IMS analysis of *Abca4* ^*−/−*^ via accurate mass and tandem MS in negative ion mode^12^. However a review of the biomedical literature to relate these findings to biological significance, indicated that isobaric bis(monoacylglycero)phosphate (BMP) lipids share exact molecular formulas with these PG lipid species^49^ (Supplement Table 1). Differentiation of structural isomers that are exact isobars such as PG and BMP lipids is challenging^50^.

### LC-MS/MS identification of BMP lipids

**Figure 4:**
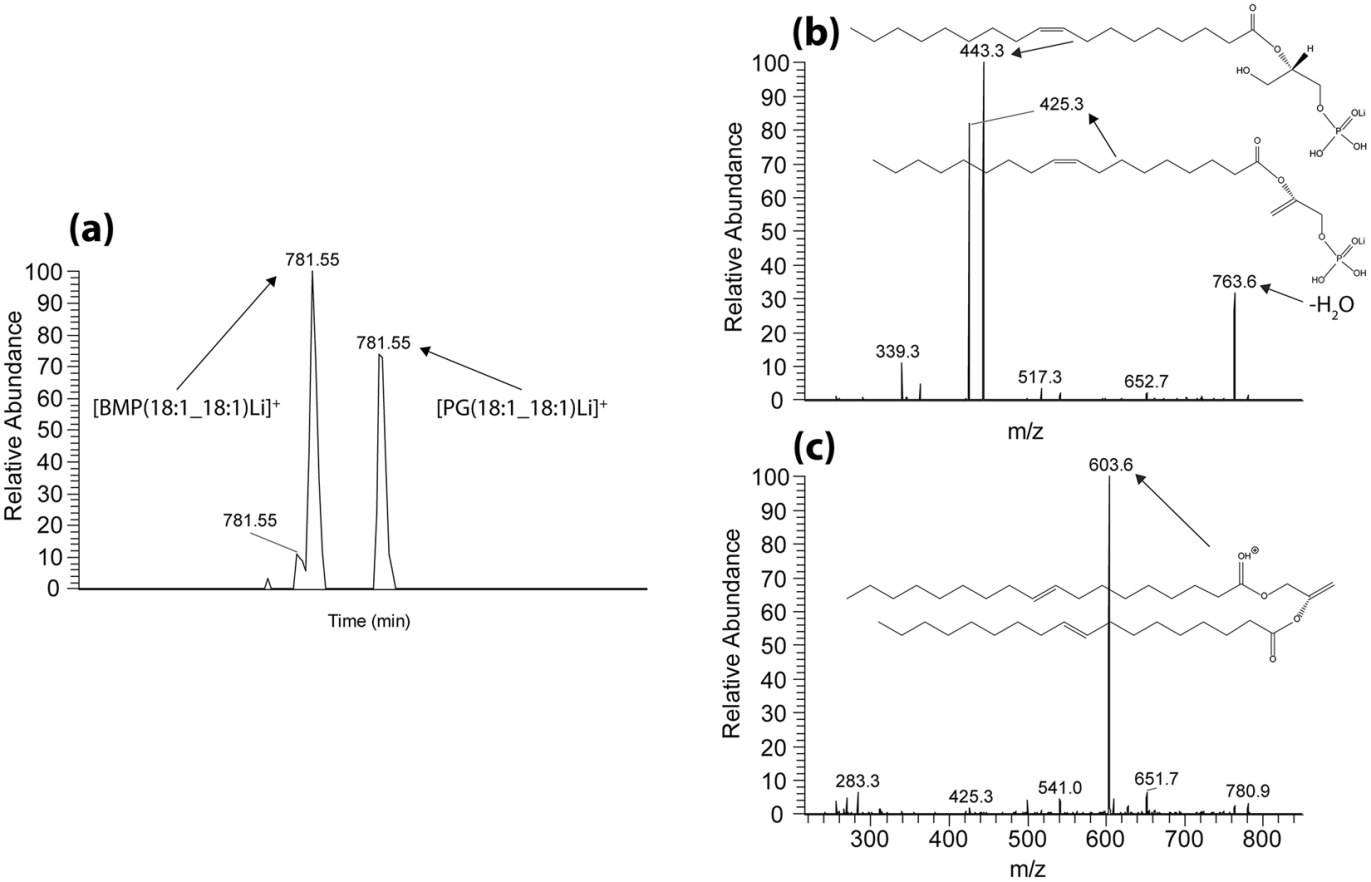
Positive ion LC-MS data from lipid standard mixture of PG(18:1/18:1) and BMP(18:1/18:1). (**a**) Extracted ion chromatogram displaying the retention times of *m/z* 781.55. (**b**) MS/MS spectrum from the peak eluting first at 21.6 minutes indicating fragments corresponding to the BMP lipid standard. (**c**) MS/MS spectrum from the peak eluting second at 22.3 minutes indicating fragments corresponding to the PG lipid species

BMP lipids represent a novel structural variation in the *sn* position of the fatty acid chains. While PG lipids have the more conventional *sn*−1, *sn*-2 orientation, BMP lipids display an unusual *sn*-1, *sn*-1 configuration^51^. This structural variation makes identification of PG and BMP lipid species via accurate mass alone impossible since they have the same exact molecular formula (Supplement Table 1). Tandem mass spectrometry (MS/MS) identification based on fragmentation patterns without standards is also challenging, since potential fatty acid losses for a given *m/z* would be the same for both PG and BMP resulting in similar fragmentation patterns. As an example, the accurate mass of *m/z* 817.503 could correspond to either PG or BMP lipids with the fatty acid side chains (18:2_22:6), (18:4_22:4), (20:3_20:5), or (20:4_20:4), to give a total of eight potential molecular entities. MS/MS analysis in negative ion mode can confirm the fatty acid chain structures, but it cannot clearly differentiate PG from BMP lipids. Therefore, PG and BMP lipid standards were analyzed using reverse phase LC-MS/MS in both negative ion mode (to identify the fatty acid chains) and positive ion mode (to confirm the *sn* configuration of the fatty acid chains)^50,52,53^. The method was optimized to ensure chromatographic separation of the two commercially available standards [PG(18:1_18:1) and BMP(18:1_18:1)] and to confirm fragmentation patterns could be used to discern the isobaric species. Analysis of standards shown in Fig. 4 showed that the BMP lipid eluted first in the chromatogram (Fig. 4a) and gave lower *m/z* fragment ions using MS/MS analysis in positive ion mode. Fragment ion signals were observed at *m/z* 443.3 and *m/z* 425.3, corresponding to fatty acid chain losses (Fig. 4b). Fragmentation of the PG standard produced a protonated fragment ion of m/z 603.6 due to loss of the head group that can be used to distinguish between PG and BMP lipids (Fig. 4c).

**Figure 5:**
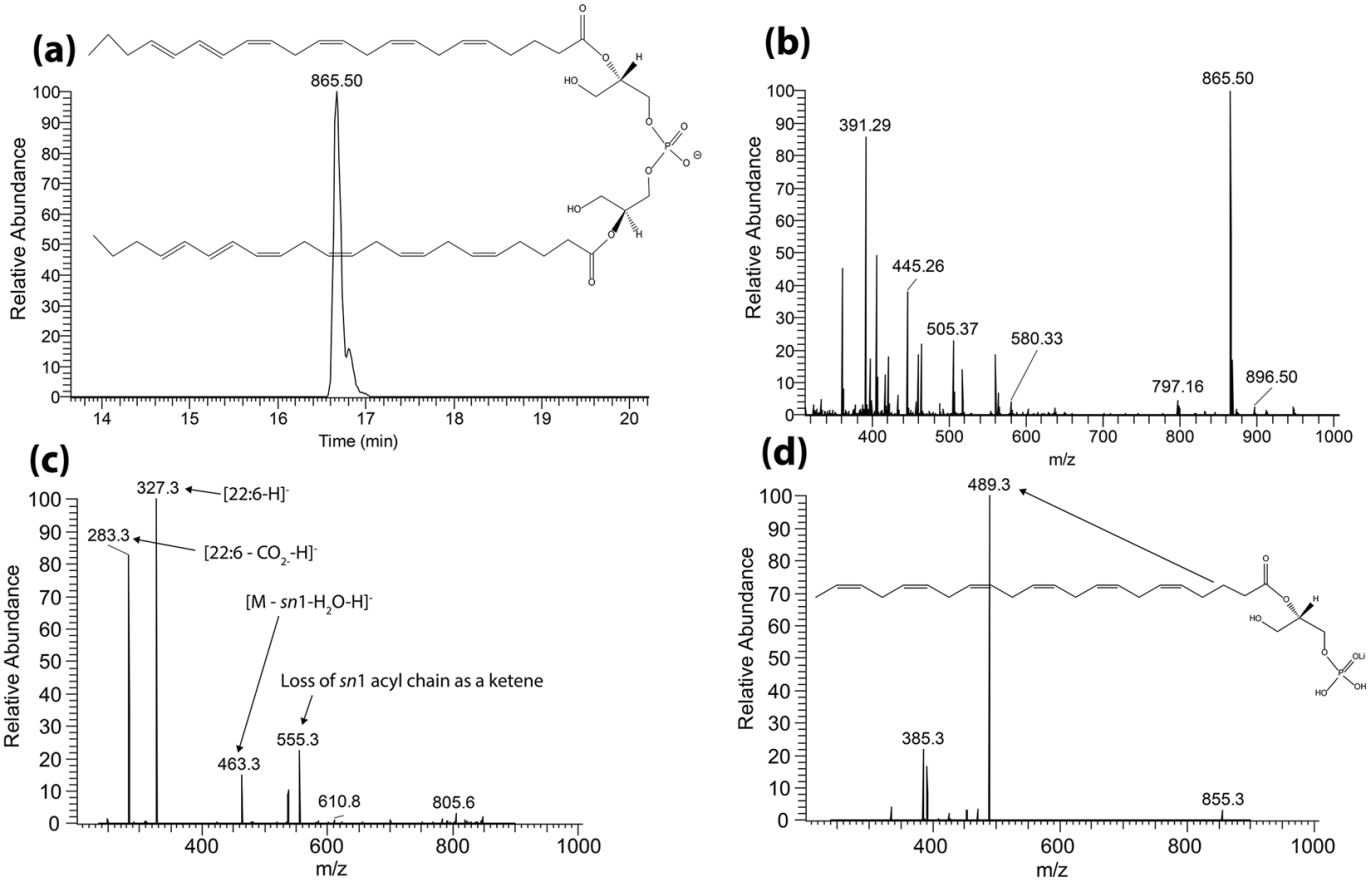
LC-MS data from *Abca4* ^*−/−*^ Folch extracted retina homogenate with corresponding structures. (**a**) Extracted ion chromatogram displaying the retention time of *m/z* 865.50. (**b**) Full scan orbitrap MS spectrum in negative ion mode from peak displayed in (**a**) showing *m/z* 865.50 as the base peak. (**c**) MS/MS mass spectrum in negative ion mode from the mass selected [M-H]^−^ ion at *m/z* 865.50. (**d**) MS/MS mass spectrum in positive ion mode from the mass selected [M + Li]^+^ adduct at *m/z* 873.5.

To decipher which lipids are responsible for the signals observed in the *Abca4* ^*−/−*^ mouse model imaging data shown in Fig. 2, targeted LC-MS/MS analysis was performed on lipid extracts from flat mounted RPE tissue from a seven month old *Abca4* ^*−/−*^ mouse eye. Figure 5 shows LC-MS data where the extracted ion chromatogram in panel (a) displays the retention time, structure and accurate mass of *m/z* 865.50 from Orbitrap analysis in full scan negative ion mode. The extracted ion chromatogram of *m/z* 865.50 is indicated in panel (a), showing a strong signal at a retention time of 16.7 minutes. Only one peak was observed suggesting the presence of only one isobaric species (either PG or BMP). The ion at *m/z* 865.50 can be seen as the base peak in the full scan FT mass spectrum at this retention time (panel b). The MS/MS spectrum in Fig. 5c displays the negative ion fragments with structures from *m/z* 865.5, indicating the 22:6 fatty acid chains at *m/z* 327.3 and loss of CO_2_ from the acyl chain at *m/z* 283.3. Positive ion mode MS/MS analysis of the lithium adduct for this species at m/z 873.53 (Fig. 5d) displays a major fragment ion at *m/z* 489.3 corresponding to loss of one of the C22:6 fatty acid chains. No evidence was observed for a fragment ion due to loss of the head group at the theoretical fragment ion mass for PG(22:6_22:6) at *m/z* 695.5 confirming the ions at *m/z* 865.50 [M-H]^-^ and *m/z* 873.53 [M + Li]^+^ correspond to BMP(22:6_22:6). Positive ion mode analysis of the lithium adducts of the two other major signals in the mass spectra extracted from retina tissue (Fig. 2), *m/z* 825.53 and *m/z* 849.53 (corresponding to [M-H]^-^ ions at *m/z* 817.50 and *m/z* 841.50) ions confirm the species observed in the RPE are BMP lipids with varying fatty acid chains. Spectra and details describing the identification of BMP(20:4_22:6) at *m/z* 841.50, BMP(20:4_20:4), and BMP(18:2_22:6) at *m/z* 817.50 can be found in Supplementary Figs 5, 6 and 7.

### BMP lipids in the human RPE

**Figure 6:**
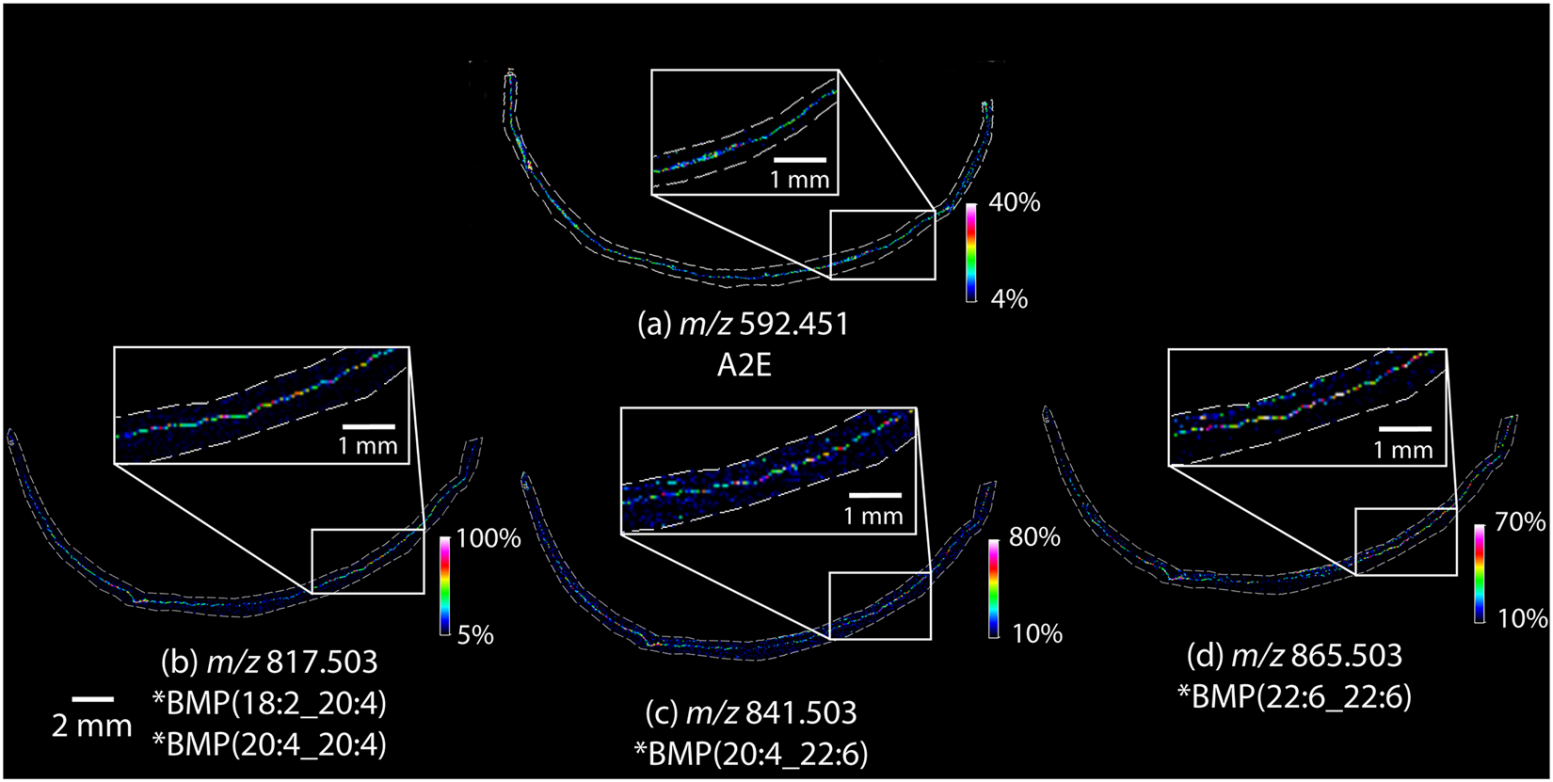
MALDI IMS analysis of 82 year old human retina tissue. (**a**) MALDI IMS data in positive ion mode displaying the relative abundance of A2E at *m/z* 592.45. MALDI IMS data in negative ion mode displaying the relative abundance of (**b**) *m/z* 817.503 (c) 841.503 and (**d**) *m/z* 865.503. Enlarged insets show zoomed in regions of RPE tissue from corresponding regions of the adjacent sections. *Identifications based on mouse tissue analysis.

MALDI imaging data taken from an entire peripheral cross section of a healthy 82 year old donor eye tissue is shown in Fig. 6. The distribution of A2E observed at *m/z* 592.451 in Panel A indicates A2E localization in the RPE layer with lower intensity pixels observed in the central areas of the tissue. Negative ion mode MALDI MS images from an adjacent section tissue section (Fig. 6b, c, and d) display the distributions the BMP lipids observed in the RPE layer of this tissue. These data were replicated from four additional adjacent sections with two analyzed in positive ion mode and two in negative ion mode (Supplementary Figs 8 and 9) and in a second 72 year old donor eye which can be seen in Supplementary Figs 11, 12 and 13. Intensity box plots displayed in Supplementary Figure 10 were generated from spectra from the RPE central region compared to spectra from the RPE in the peripheral region from the section analyzed in Fig. 6, indicating how the ion intensity of the BMP lipids changes in these regions.

## Discussion

High mass resolution MALDI IMS of animal model tissue provided the localization of 3 signals that were uniquely present in the RPE layer of *Abca4* ^*−/−*^ mouse tissue when compared to *Sv129* control. These findings were confirmed in multiple replicates from three separate animals. A number of other bis-retinoids^54^, previously observed to vary in abundance between the *Abca4* ^*−/−*^ and control strains, were not observed as they might not be amenable to the MALDI ionization process. The signals observed with high abundance in the *Abca4* ^*−/−*^ mouse model in these data were distinguished from potential isobaric species and identified using LC-MS/MS analysis in both positive and negative ionization mode. This identification method revealed that the lipids responsible for the signals of interest were BMP lipids. Images obtained from a peripheral section of a human eye display A2E localized to the RPE with similar intensity observed across the breath of the section. The *m/z* values corresponding to BMP lipid species identified in the *Abca4* ^*−/−*^ are also observed in the RPE across the breadth of the human tissue sections. Previously described as a ‘peculiar phospholipid’^49^, BMP lipids have been implicated in a number of conditions involving late endosomal/lysosomal dysfunction. Specifically, the accumulation of BMP lipids in late endosomes and lysosomes has been reported in Niemann-Pick (NP) disease^46,48^, neuronal ceroid lipofuscinosis (NPC)^47^, and as a result of drug induced phospholipidosis^49,55^.

The pathways leading to the formation of BMP lipids are not well understood. In drug-induced phospholipidosis it is thought that they accumulate either due to interactions of cationic amphiphilic drugs with endogenous lipids to produce indigestible metabolites or due to the direct inhibition of the enzymes involved in lipid recycling and degradation^49^. Since A2E is known to accumulate in the lysosomes and is both cationic and amphiphilic in nature and, since its precursor A2-PE is formed from retinaldehyde and phosphatidylethanolamine (a modified lipid^14^), the formation of BMPs in the Abca4^*−/−*^ mouse is likely due to pathways similar to those triggered by cationic amphiphilic drugs^56^. The inhibition of lipid degradation and of phagocytosed photoreceptor outer segments, and the accumulation of free and esterified cholesterol caused by the addition of A2E at physiological concentrations to RPE cell cultures have been previously demonstrated^19,20,57^. Moreover, there is clear evidence from multiple studies that BMP lipids regulate cellular cholesterol content in the endosome-lysosome compartment^48,58–60^.

The presence of cholesterol is essential for the formation of cell membranes, synapses, and dendrites in the neuronal retina; however, little is known about mechanisms that regulate cholesterol homeostasis in retinal tissue^60,61^.

Interestingly, the fatty acid constituents of BMP lipids observed herein were mainly polyunsaturated fatty acids such as docosahexaenoic acid (22:6), a fatty acid known to be highly abundant in photoreceptors and to be crucial in photoreceptor cell recycling and survival^62–64^. BMP lipids are known to have a propensity to incorporate DHA preferentially over other fatty acids and to be resilient to phospholipases^49^. Sequestration of DHA in the endosomes in the form of BMP lipids may prevent its recycling and prevent production of neuroprotectin D1, as neuroprotecin D1 has been shown to prevent oxidative stress induced apoptosis of RPE cells this sequestration may have a detrimental effect on the RPE cells^62,65^. Another interesting aspect of polyunsaturated lipids BMP lipids is there potential to function as anti-oxidants as described by Hullin-Matsuda *et al*., they suggest that BMP(22:6/22:6) could exert anti-oxidant action towards cholesterol that transits through the late endosomes as BMP(22:6/22:6) is highly prone to peroxidation^49^. Nevertheless, the relevance of the fatty acid composition of BMP lipids in relation to cellular functions in the RPE tissue remains an active area of investigation.

Inhibition of phospholipases and disruption of cholesterol trafficking or formation of oxysterols in the RPE may help explain the accumulation of cholesterol in the drusen deposits under the RPE^19,20,66,67^, particularly in the periphery, where the bulk of A2E is found in the human eye^11,23,44^.

In conclusion, MALDI IMS combined with LC-MS/MS analysis has established the presence of BMP lipids in the Abca4^*−/−*^ mouse and in human retina tissues. Lack of the ABCA4 protein, a Stargardt disease locus, leads to the accumulation of BMP lipids, known to be elevated in endosomal/lysosomal storage diseases and is associated with impaired late endosomal/lysosomal lipid processing. These results suggest that the pathological mechanisms underlying Stargardt disease may be similar to those in endosomal/lysosomal storage disorders. The presence of BMP lipids in the human retina is intriguing and requires further investigation of endosomal/lysosomal function and age-related changes in this tissue.

## Methods

### Animal and human tissues

*Abca4* ^*−/−*^ mice were obtained from established colonies at the Medical University of South Carolina that originated from breeding pairs generously provided by Dr. G.H. Travis (Jules Stein Eye Institute, University of California – Los Angeles). The background strain was *Sv129*. Animals were reared in cyclic light with a 12-h light cycle (06:00–18:00). All animal procedures were carried out in accordance with protocols approved by the Institutional Animal Care and Use Committee of the Medical University of South Carolina and were consistent with the recommendations of the Panel on Euthanasia of the American Veterinary Medical Association. Six month old animals were dark-adapted overnight, sacrificed under dim red light, and the eyes were removed. Frozen eyes were shipped on dry ice from Charleston to Nashville for experiments. A human cadaver eye (age 72 and 82 years, 24–48 hours postmortem) was obtained from the San Diego Eye Bank (San Diego, CA). The human tissue used on our studies described in our manuscript was obtained from deceased human donors, therefore considered non-human subject research. The samples were embedded as described below, in Charleston and the frozen embedded sample was shipped on dry ice to Nashville for analysis.

### Preparation of tissues for MALDI IMS

Whole mouse eyes from three six month old *Abca4* ^*−/−*^ (based on the *Sv129* background strain) and three *Sv129* animals were embedded by placing the frozen eye in 1% carboxymethylcellulose^68^ (Sigma Aldrich, St. Louis, MO, USA) and rapidly freezing the media by placing the eye into a basin constructed from Parafilm (Fisher Scientific, GA, USA) dry ice. Three adjacent 12 µm thick sections were obtained with a cryostat (Leica CM3050S, IL, USA) at -20 °C and thaw mounted onto a gold coated MALDI target plate (AB Sciex, Ontario, Canada). The sections were dehydrated in a vacuum desiccator for 60 minutes For positive mode analysis, 2,5-Dihydroxyacetophenone (DHA) (Sigma Aldrich, St. Louis, MO, USA) was sublimated^69^ onto the samples using an in-house sublimation apparatus for eight minutes at approximately 56 mTorr and 110 °C. 1,5-Diaminonaphthalene (DAN) (Sigma Aldrich, St. Louis, MO, USA) was sublimated for 15 minutes under the same conditions for negative mode analysis.

Human retina tissue experiments were performed using 72 and 82 year old donor eyes. The tissue was prepared by removing the lens and drawing out the vitreous humor with a pipette, and replacing it with carboxymethyl cellulose (CMC, 1.0% in water for the 82 year old and 2.6% CMC for the 72 year old). The tissue and CMC then were frozen using similar methods as described above, substituting liquid nitrogen for dry ice, to preserve the native structure and shape of the remaining eye cup. Six sagittal sections, 12 μm thick across the entire diameter of the eye cup, were collected and placed onto gold-coated MALDI target plates (AB Sciex, Concord, Ontario, Canada). Once thaw mounted, the plates were dried in a vacuum desiccator for 60 minutes. MALDI matrices were applied as described above.

### MALDI IMS analysis

High mass resolution imaging experiments were performed using a Bruker 15 T or a 9.4 T solariX FTICR mass spectrometer (Bruker Daltonics, Billerica, MA, USA). The instrument is equipped with a modified smartbeam laser (Bruker Daltonics) that produces a 355 nm Gaussian beam laser profile operated at 2 kHz^70^. The laser spot size and repetition rate is tunable through the instrument software. The laser was set to the minimum spot size (~10 μm) and the x/y coordinate pitch was set to 15 μm and 50 μm pixel size for rodent and human tissue images, respectively. Mouse retina images were acquired with an average of 100 laser shots/pixel over a mass range of *m/z* 400–2000 in both positive and negative ion modes. Data from human tissue analyzed in positive ion mode was collected using 500 shots per pixel over a mass range of *m/z* 506–2000. To maximize sensitivity of human tissue negative ion mode experiments, continuous accumulation of selected ions (CASI) was used to isolate and enrich target ions. The experiment was performed by isolating a wide window (45 Da) around *m/z* 855. Selected ions produced from 3000 laser shots were then stored and accumulated (maximizing sensitivity for the selected m/z window) prior to transfer to the ICR cell for analysis. This process is applied to all pixels in the CASI FTICR imaging experiment. All experiments were performed in triplicate to minimize technical variability. Data from a 82 year old donor were acquired on a 15 T FTICR instrument, data from a 72 year old donor were acquired on a 9.4 T FTICR instrument, Images were generated using FlexImaging 4.0 (Bruker Daltonics, Billerica, MA, USA) and data were normalized to the total ion current. Intensity box plots were generated using SCiLS Lab 2018a (SCiLS, Bremen, Germany). External calibration was performed using Agilent Technologies Inc (Santa Clara, CA, USA) ESI-L low concentration tune mix in electrospray mode prior to imaging data acquisition. All FTICR data produced for negative ion studies had mass resolving powers from 93313–118849 at m/z 865.50 and mass accuracies <1 ppm. All 9.4 T data produced for negative ion studies had mass resolving powers from 62399–65594 with mass accuracies <6 ppm.

Replicate data from mouse tissue were acquired from two separate *Abca4* ^*−/−*^ and *Sv129* animals using a Bruker Rapiflex Tissuetyper equipped with a smartbeam™ 3D laser. Focus and laser power settings were optimized for 10 μm pixel size with 300 shots per pixel; beam scan was not enabled. Calibration was performed externally with a series of phosphorus clusters prior to imaging data acquisition^71^ and internally, following data acquisition, using highly abundant endogenous lipids identified from FTICR experiments.

### LC-MS/MS Analysis

#### Standards

1,2-Dioleoyl-*sn-*glycero-3-[phospho-*rac*-(1-glycerol)], PG(18:1_18:1), 25 mg sodium salt) and sn-(3-Oleoyl-2-hydroxy)-glycerol-1-phospho-sn-1′-(3′-oleoyl-2′-hydroxy)-glycerol, BMP(18:1_18:1), 5 mg ammonium salt) standards were obtained from Avanti Polar Lipids Inc. (Avanti Polar Lipids Inc. Alabaster, AL, USA). Stock solutions were made by adding 1 mL of 2:1 chloroform: methanol to the vial provided (resulting concentrations: PG 32.28 mM and BMP 6.4 mM) dilutions were performed using methanol to obtain a 200 nM stock for each and stock solutions were combined to give a final lipid concentration of 100 nM. LC-MS/MS analysis was performed on a LTQ Orbitrap XL instrument equipped with an IonMax electrospray source with a 50 μm ID stainless steel capillary (Thermo Fisher Scientific, Waltham, MA, USA). The Orbitrap was calibrated in negative ion mode using SDS, taurocholic acid, and Ultramark 1621 (LTQ negative ion calibration solution, Thermo Scientific Pierce) and in positive ion mode using caffeine, MRFA, and Ultramark 1621 (LTQ positive ion calibration solution, Thermo Scientific Pierce). HPLC separation was achieved using a Waters UPLC Acquity system at a flow rate of 0.3 mL/min, 1 M ammonium acetate (pH ~6) in methanol was added to give a 10 mM concentration in mobile phase A (H_2_O:acetonitrile:methanol 3:1:1) and mobile phase B (acetonitrile:isopropanol), (1:1). The LC gradient had a total run time of 30 minutes starting at 60% A and ramping to 100% B over a 25-minute period. 10 µL of the 100 nM standard mixture was loaded onto a Waters Acquity HSS C18 reverse-phase column (2.1 mm × 150 mm, 1.7μm) and a blank methanol injection was made between each run to minimize carryover. Negative ion data were acquired to confirm the fatty acid chain composition. Full scan spectra with a mass range of *m/z* 215–850 (resolution 60,000 at m/z 400) using the Orbitrap were acquired as the initial scan event with product ion scanning (CE = 30%) in the ion trap of the [M-H]^−^ precursor ion *m/z* 773.5 (theoretical [M-H]^−^ of the standard, *m/z* 773.534). To enhance the sensitivity of electrospray ionization in positive mode, lithium trifluoroacetate was added to both mobile phases A and B to a final concentration of 0.1 mM. Full scan MS spectra were acquired using the orbitrap (*m/z* 215–850) and product ion scanning was performed on the [M + Li]^+^ precursor ion *m/z* 781.5 (theoretical [M + Li]^+^ of the standard, *m/z* 781.557).

### LC-MS tissue experiments

To enable targeted lipid extraction specifically from the RPE layer, the neuronal retinal was removed from flat mounted mouse tissue leaving only the RPE and sclera. The tissue was embedded in a small amount of deionized water (∼250 μL) prior to mounting to the cryostat sample stage using OCT to allow for sectioning without OCT contamination. The ice also acted as a support material so the tissue could be sectioned along its longest axis. The tissue was then sectioned at 3 µm increments along the full length (longest axis) using a Leica CM3050S cryostat (Leica CM3050S, IL, USA). Sections were placed into a silanized clear glass vial (National Scientific, TN, USA) with 1 mL of chloroform:methanol (2:1). The samples were vortex-mixed for three minutes. Deionized water (2 × 200 µL) was added and vortex mixed for 2 minutes each time. The solution was then allowed to separate into the two phases before the upper phase was removed and discarded as waste. The interface was then rinsed (no mixing) with chloroform:methanol:water (3:48:47) 3× siphoning 200 µL of the upper phase each time and discarding to waste. The remaining lower phase was then dried down using a stream of dehumidified nitrogen. 100 µL of 100% methanol was used to reconstitute the sample which was extensively vortex mixed before microcentrifugation at 12000 rpm for five minutes at 4 °C to pellet any particulate. A 40 µL sample was placed into a 250 μL silanized glass polyspring insert in a 2-mL glass autosampler vial. LC-MS/MS experiments were performed using the method described above. Negative ion analysis was performed with in the orbitrap full scan MS over a mass range of *m/z* 200–1000 with targeted product ion scanning for precursor ions *m/z* 817.5, *m/z* 841.5, and *m/z* 865.5. Positive ion analysis was performed with full scan MS spectra over a mass range of *m/z* 200–1000 and product ion scanning (CE = 30%) of the [M + Li]^+^ precursor ions of the species of interest (*m/z* 825.5, 849.5 and 873.5).

## Electronic supplementary material

Supplementary Information
