# Supplementary material for: Bis(monoacylglycero)phosphate lipids in the retinal pigment epithelium implicate lysosomal/endosomal dysfunction in a model of Stargardt disease and human retinas
Source: Sci Rep. 2017 Dec 11;7:17352. doi: 10.1038/s41598-017-17402-1 (PMC5725462; doi:10.1038/s41598-017-17402-1)
Supplement: Supplementary file 1 — Supplementary Information [file 41598_2017_17402_MOESM1_ESM.pdf]

# Supplementary Information

## Bis(monoacylglycero)phosphate lipids in the retinal pigment epithelium implicate lysosomal/endosomal dysfunction in a model of Stargardt disease and human retinas.

**David M. G. Anderson, Zsolt Ablonczy, Yiannis Koutalos, Anne M. Hanneken, Jeffrey M. Spraggins, Wade Calcutt, Rosalie K. Crouch, Richard M. Caprioli, Kevin L. Schey.**

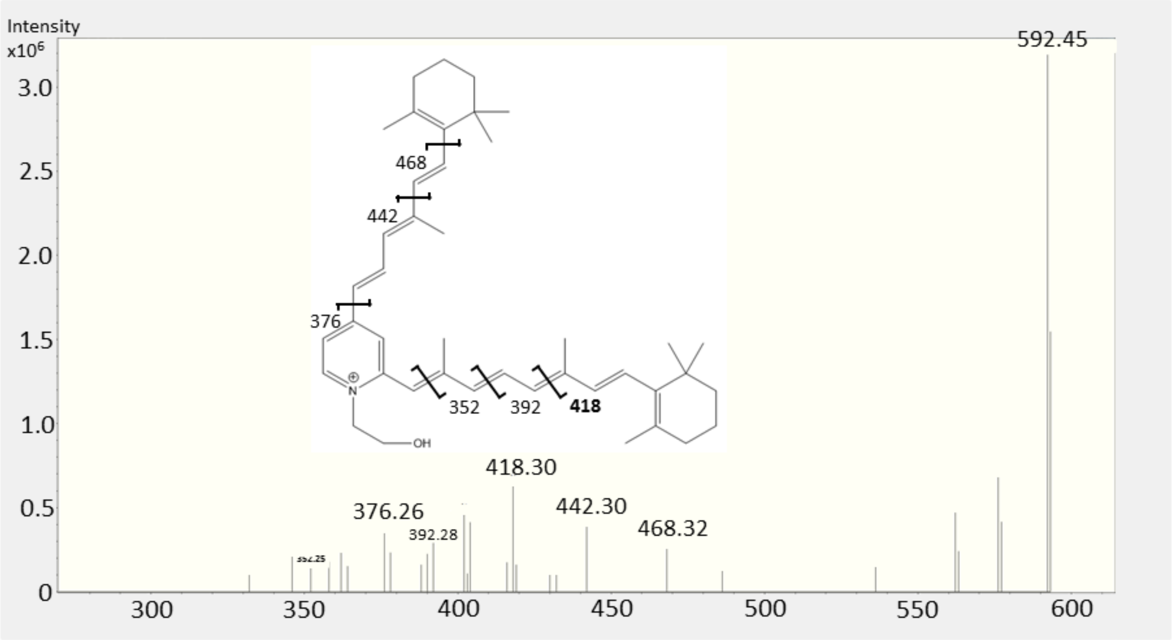


Supplementary figure 1. MS/MS spectrum of A2E standard

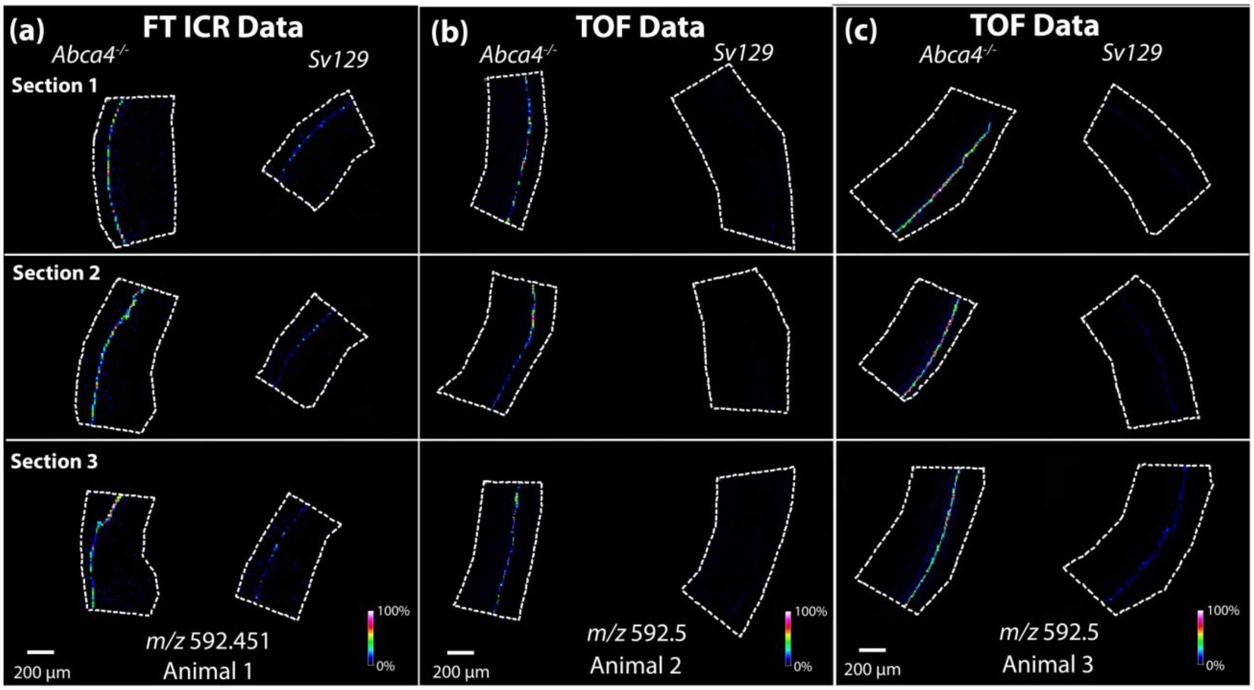


Supplementary figure 2. (a) FTICR analysis of replicates from animal 1 displaying the distribution of A2E at *m/z* 592.451. (b) TOF analysis of replicates displaying the distribution of A2E at *m/z* 592.5 from animal 2. (c) TOF analysis of replicates displaying the distribution of A2E at *m/z* 592.5 from animal 3.

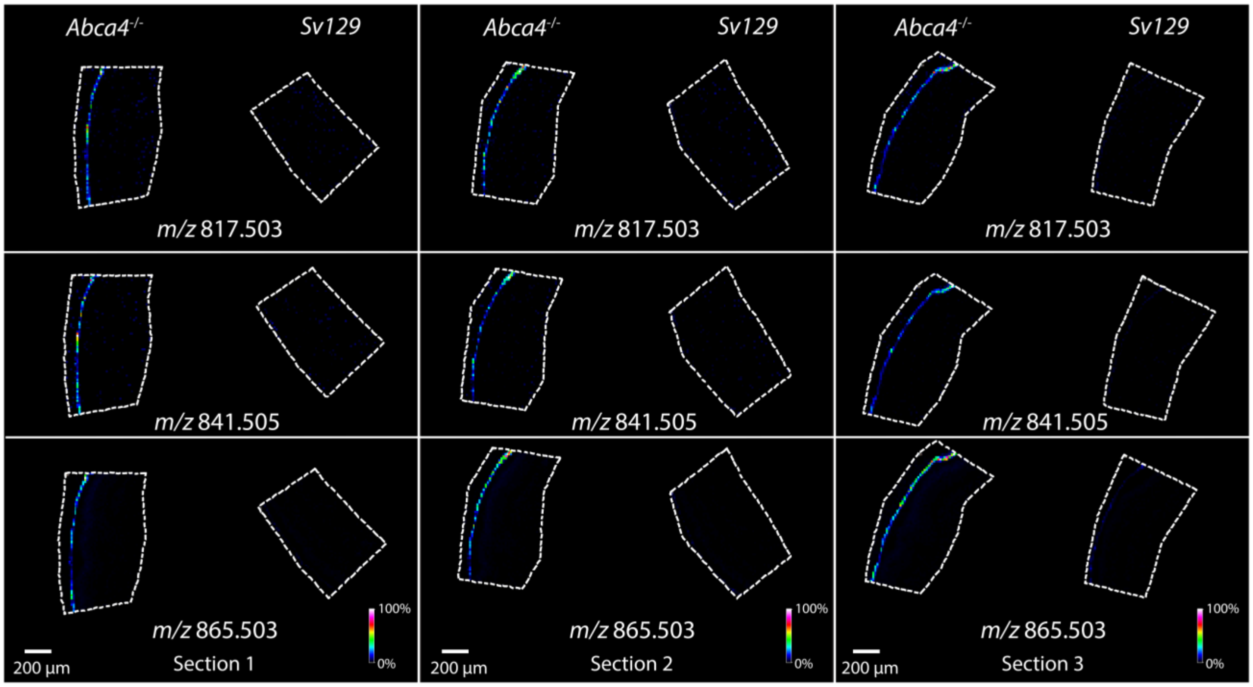


Supplementary figure 3. FTICR analysis of replicates from animal 1 displaying the distribution of *m/z* 817.503, *m/z* 841.503, and *m/z* 865.503 from three sections from animal 1.

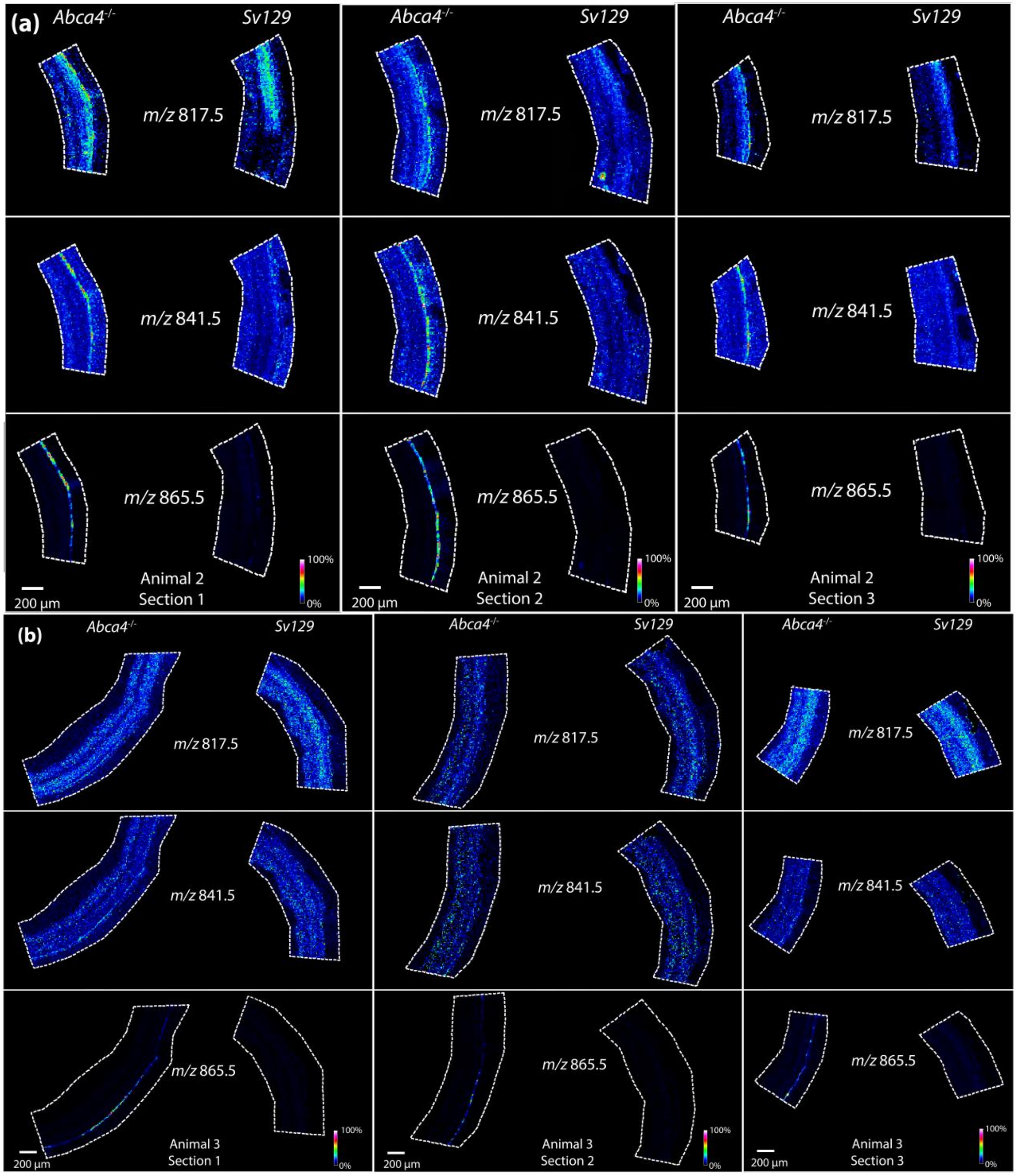


Supplementary figure 4. TOF analysis of replicates from (a) animal 2 and (b) animal 3 displaying the distribution of *m/z* 817.5, *m/z* 841.5, and *m/z* 865.5.

| Molecular species | Molecular Formula | Molecular weight | Theoretical $M^+$ Ion | Theoretical $M\text{-}H^-$ Ion |
|---|---|---|---|---|
| A2E | $C_{42}H_{58}NO^+$ | 592.451 | 592.45129 | n/a |
| A2GPE | $C_{45}H_{65}NO_6P^+$ | 746.45549 | 746.45549 | n/a |
| BMP(18:2_20:4 | $C_{46}H_{75}O_{10}P$ | 818.50924 | n/a | 817.50251 |
| BMP(20:4_20:4) | $C_{46}H_{75}O_{10}P$ | 818.50924 | n/a | 817.50251 |
| BMP(20:4_22:6) | $C_{48}H_{75}O_{10}P$ | 842.50924 | n/a | 841.50251 |
| BMP(22:6_22:6) | $C_{50}H_{75}OP$ | 865.50924 | n/a | 865.50251 |
| PG(18:2_22:6) | $C_{46}H_{75}O_{10}P$ | 818.50924 | n/a | 817.50251 |
| PG(20:4_20:4) | $C_{46}H_{75}O_{10}P$ | 818.50924 | n/a | 817.50251 |
| PG(20:4_22:6) | $C_{48}H_{75}O_{10}P$ | 842.50924 | n/a | 841.50251 |
| PG(22:6_22:6) | $C_{50}H_{75}OP$ | 865.50924 | n/a | 865.50251 |

Supplementary Table 1.

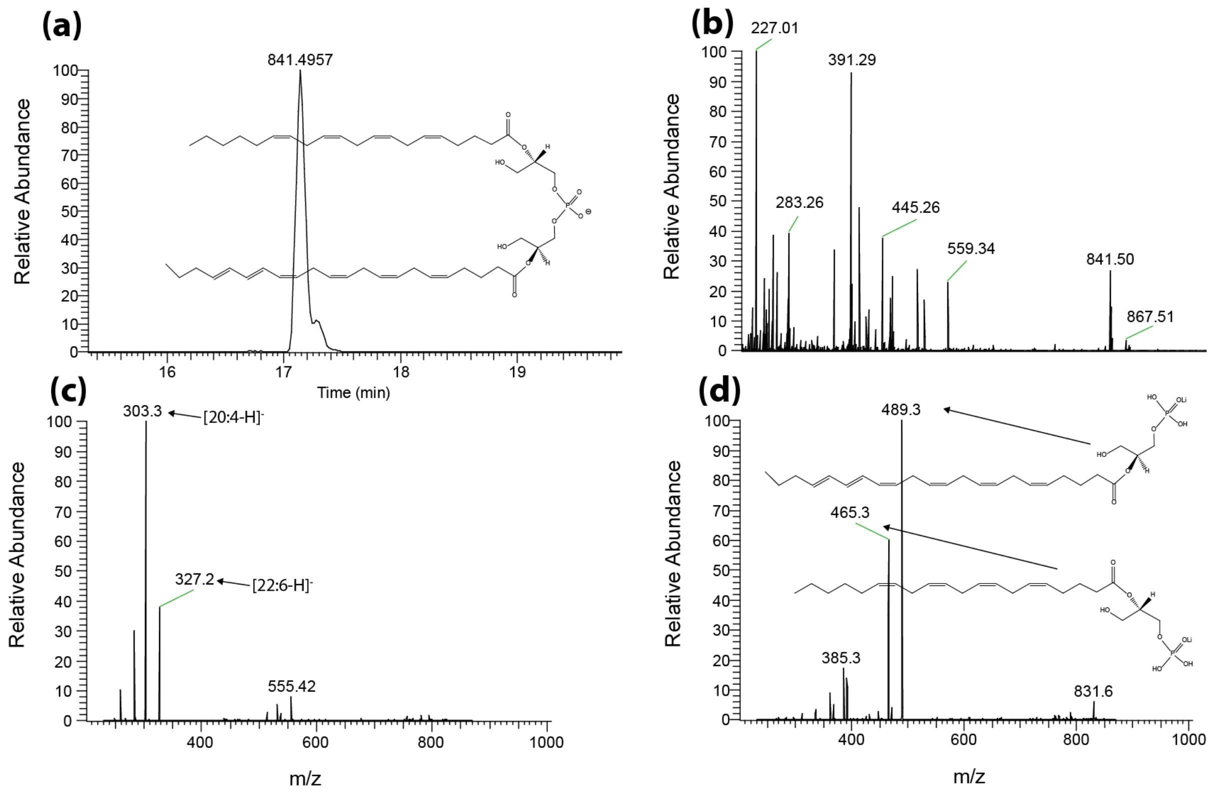


Supplementary figure 5. LC-MS data from *Abca4*$^{-/-}$ Folch extracted retina homogenate. (a) Extracted ion chromatogram displaying the retention time of *m/z* 841.50 at 17.13 minutes. (b) Full scan orbitrap MS spectrum from peak displayed in (a) showing *m/z* 841.5. (c) MS/MS mass spectrum in negative ion mode from the mass selected $[M-H]^-$ ion at *m/z* 841.50. (d) MS/MS mass spectrum in positive ion mode from the mass selected $[M+Li]^+$ adduct at *m/z* 849.5.

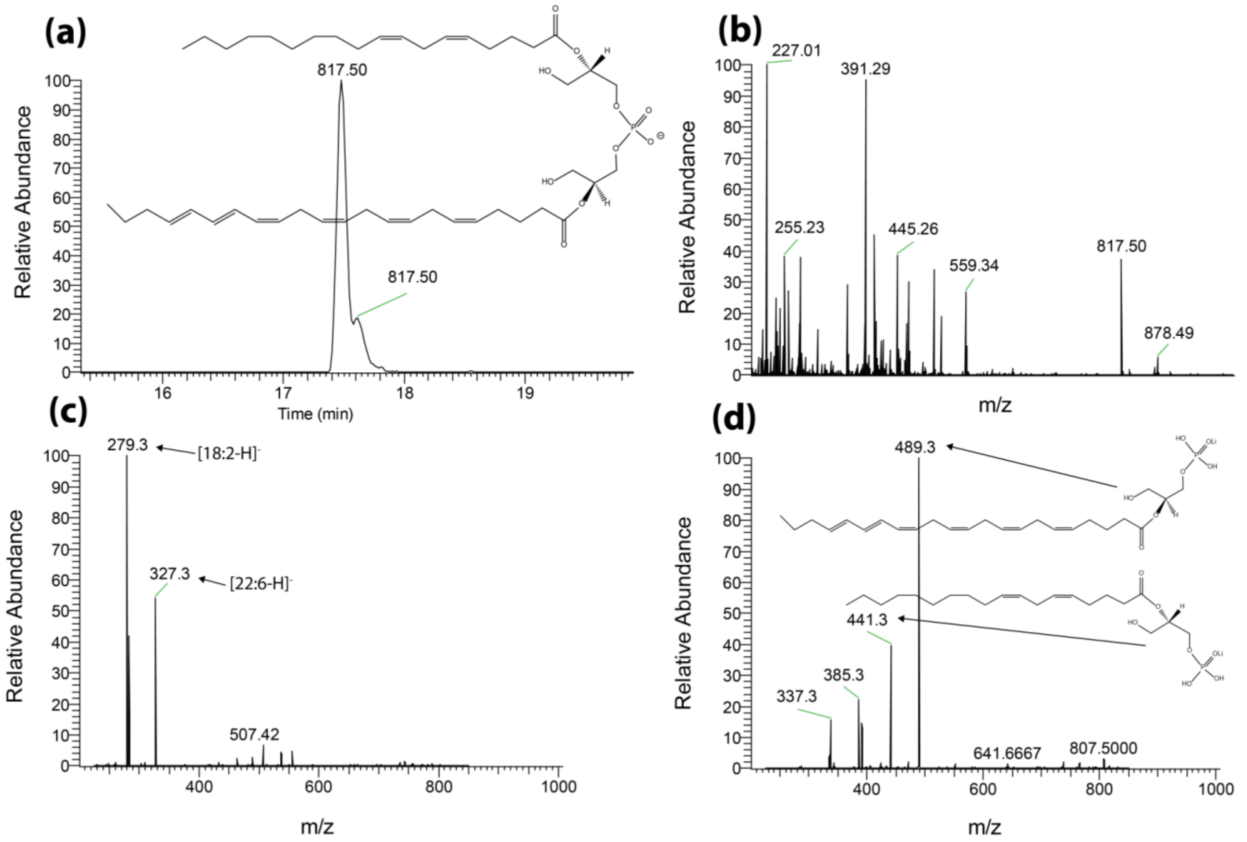


Supplementary figure 6. LC-MS data from *Abca4*$^{-/-}$ Folch extracted retina homogenate with corresponding structures. (a) LC-MS chromatogram displaying the retention time of *m/z* 817.50. (b) Full scan orbitrap MS spectrum from peak displayed in (a) showing *m/z* 817.5. (c) MS/MS mass spectrum in negative ion mode from the mass selected $[M-H]^-$ ion at *m/z* 817.50. Spectrum summed from the main peak observed in the chromatogram in panel (a). (d) MS/MS mass spectrum in positive ion mode from the mass selected $[M+Li]^+$ adduct at *m/z* 825.5. Spectrum summed from the main peak (not shoulder) at 17.45 minutes observed in the chromatogram in panel (a).

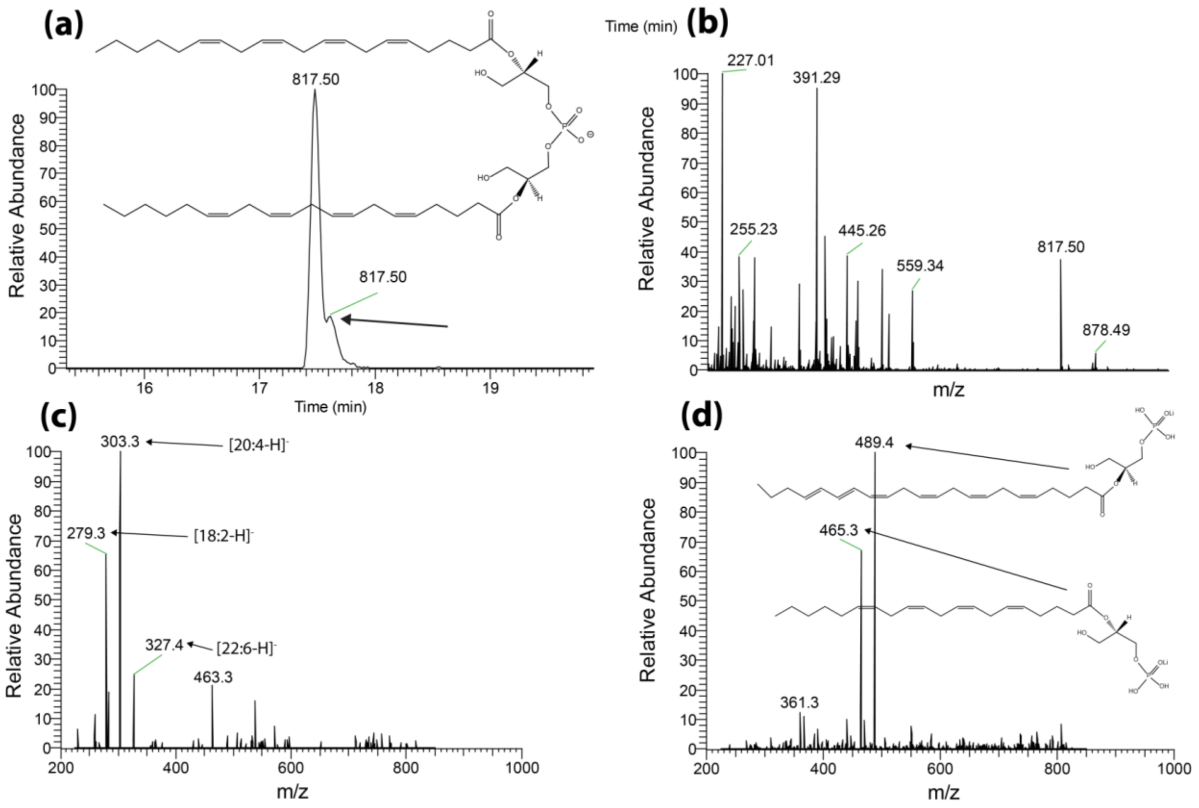


Supplementary figure 7. LC-MS data from *Abca4*$^{-/-}$ Folch extracted retina homogenate with corresponding structures. (a) LC-MS chromatogram displaying the retention time of *m/z* 817.50. (b) Full scan orbitrapMS spectrum from peak displayed in (a) showing *m/z* 817.5. (c) MS/MS mass spectrum in negative ion mode from the mass selected $[M-H]^-$ ion at *m/z* 817.50. Spectrum summed from the shoulder peak observed in the chromatogram in panel (a), indicated by the arrow. (d) MS/MS mass spectrum in positive ion mode from the mass selected $[M+Li]^+$ adduct at *m/z* 825.5. Spectrum summed from the shoulder peak observed in the chromatogram at 17.62 minutes indicated by the arrow panel (a).

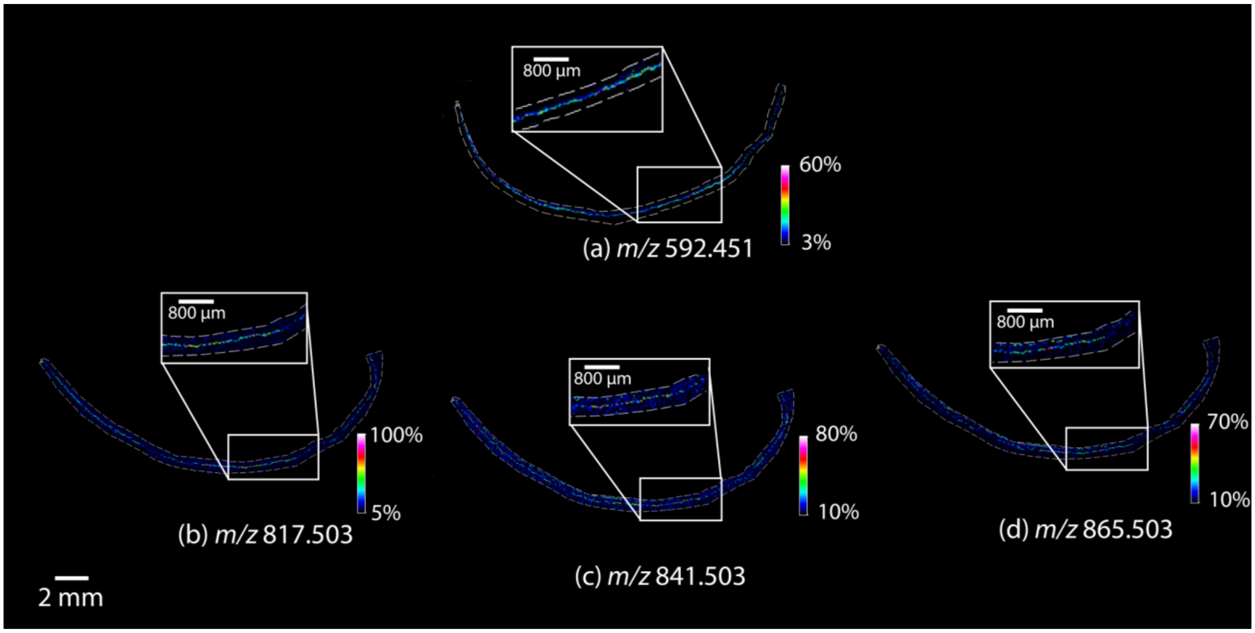


Supplementary Figure 8. MALDI IMS analysis of 82 year old human retina tissue from sections adjacent to those observed in Figure 5 or supplement figure 9. (a) MALDI IMS data in positive ion mode displaying the relative abundance of *m/z* 592.451. MALDI IMS data in negative ion mode displaying the relative abundance of (b) *m/z* 817.503 (c) 841.503 and (d) *m/z* 865.503. Enlarged insets show zoomed in regions of RPE tissue from corresponding regions of the adjacent sections.

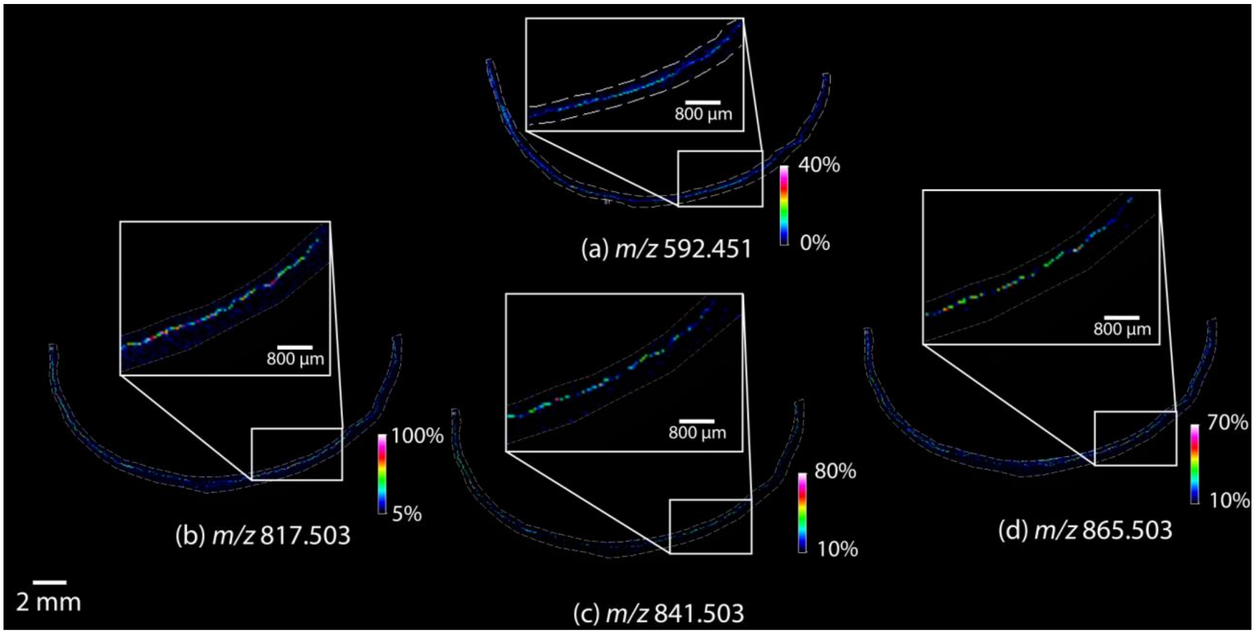


Supplementary Figure 9. MALDI IMS analysis of 82 year old human retina tissue from sections adjacent to those observed in Figure 5 or supplement figure 8. (a) MALDI IMS data in positive ion mode displaying the relative abundance of *m/z* 592.45. MALDI IMS data in negative ion mode displaying the relative abundance of (b) *m/z* 817.503 (c) 841.503 and (d) *m/z* 865.503. Enlarged insets show zoomed in regions of RPE tissue from corresponding regions of the adjacent sections.

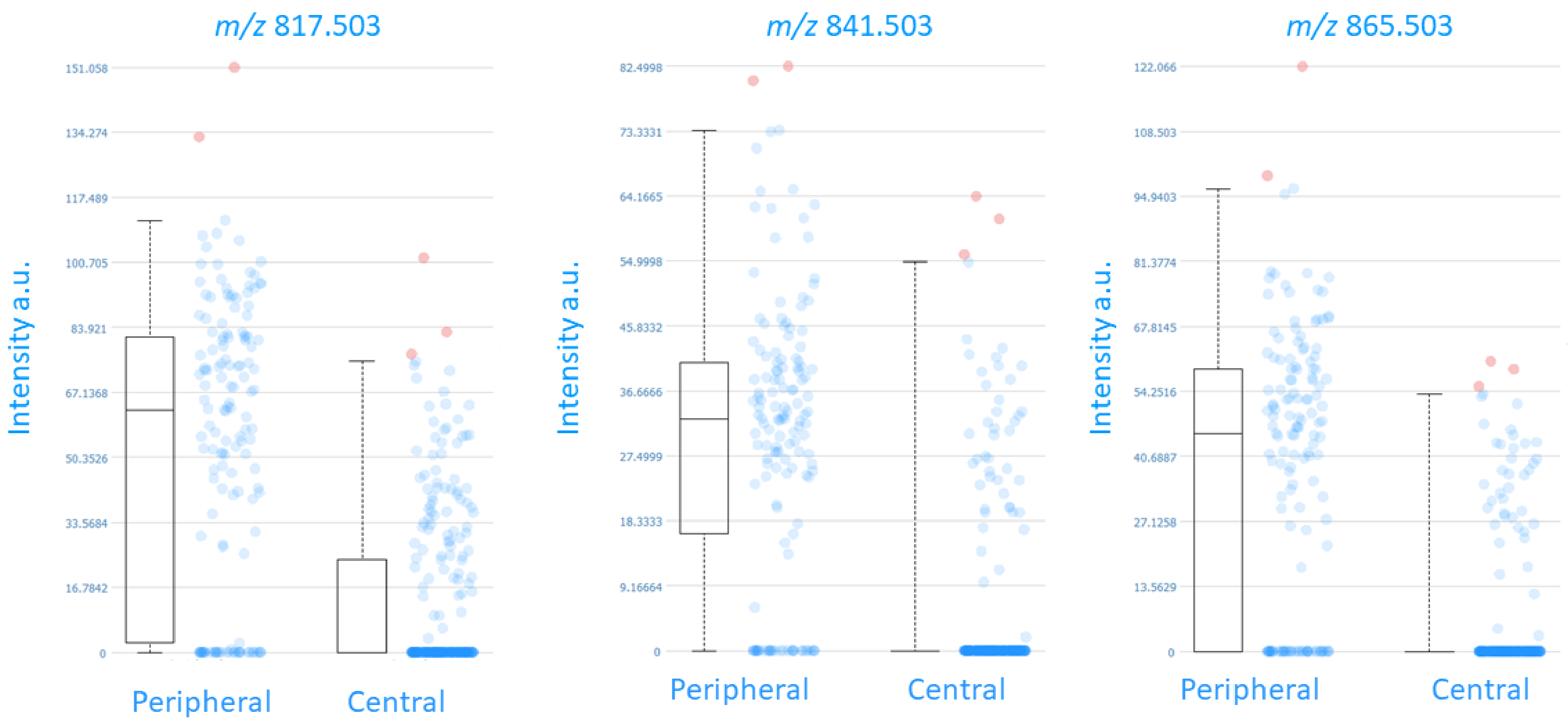


Supplementary figure 10. Intensity box plots derived from spectra in the central and peripheral regions of the RPE tissue from the MALDI IMS data of the 82 year old donor retinal section analyzed in figure 6. The graphs indicate the relative intensities of the specified *m/z* values in peripheral and central regions of the RPE tissue in the section. The average intensities in peripheral regions for *m/z* 817.503, 841.503 and 865.503 are indicated by line in the bar. The blue and red data points represent values from individual pixels within the image. Data points or pixels included in the regions selected with zero intensity reside on the zero line.

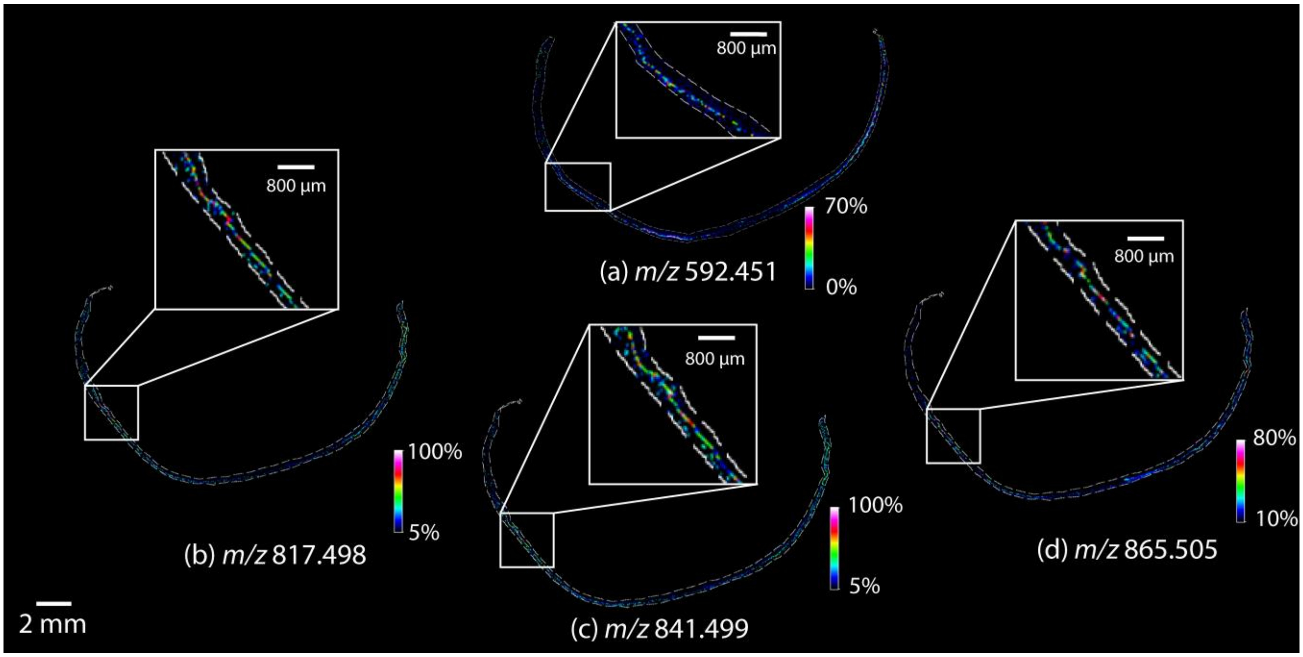


Supplementary figure 11. MALDI IMS analysis of 72 year old human retina tissue. (a) MALDI IMS data in positive ion mode displaying the relative abundance of *m/z* 592.451. MALDI IMS data in negative ion mode displaying the relative abundance of (b) *m/z* 817.498 (c) 841.499 and (d) *m/z* 865.505. Enlarged insets show zoomed in regions of RPE tissue from corresponding regions of the adjacent sections.

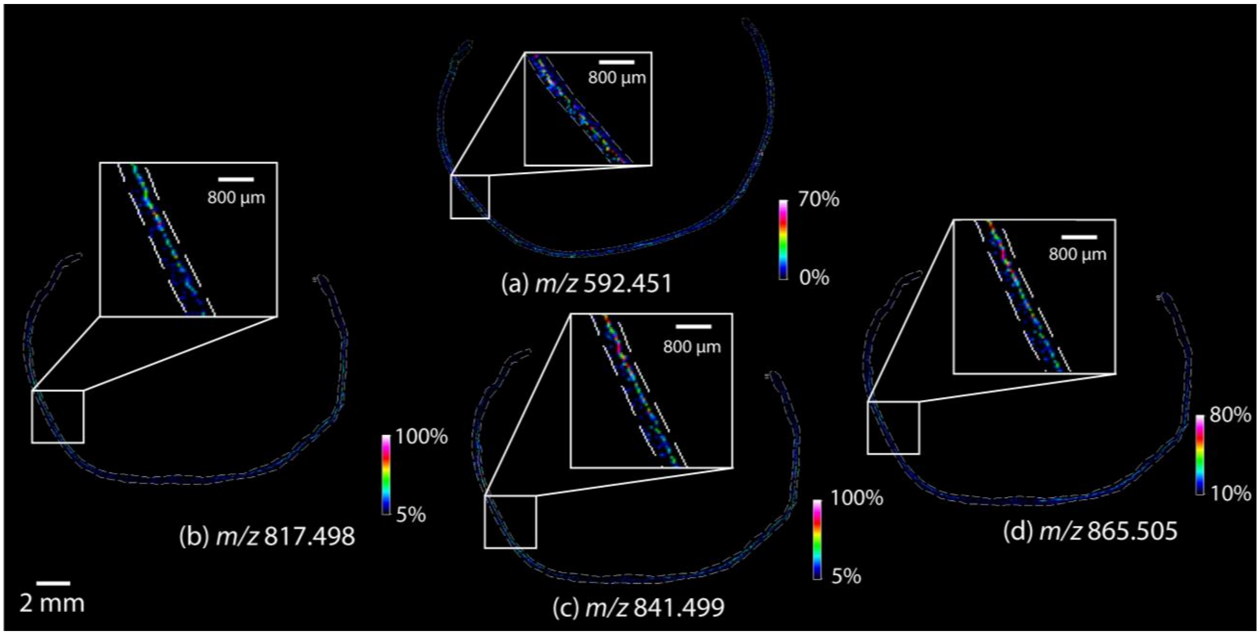


Supplementary figure 12. MALDI IMS analysis of 72 year old human retina tissue from sections adjacent to those in supplementary fig 10. (a) MALDI IMS data in positive ion mode displaying the relative abundance of *m/z* 592.451. MALDI IMS data in negative ion mode displaying the relative abundance of (b) *m/z* 817.498 (c) 841.499 and (d) *m/z* 865.505. Enlarged insets show zoomed in regions of RPE tissue from corresponding regions of the adjacent sections.

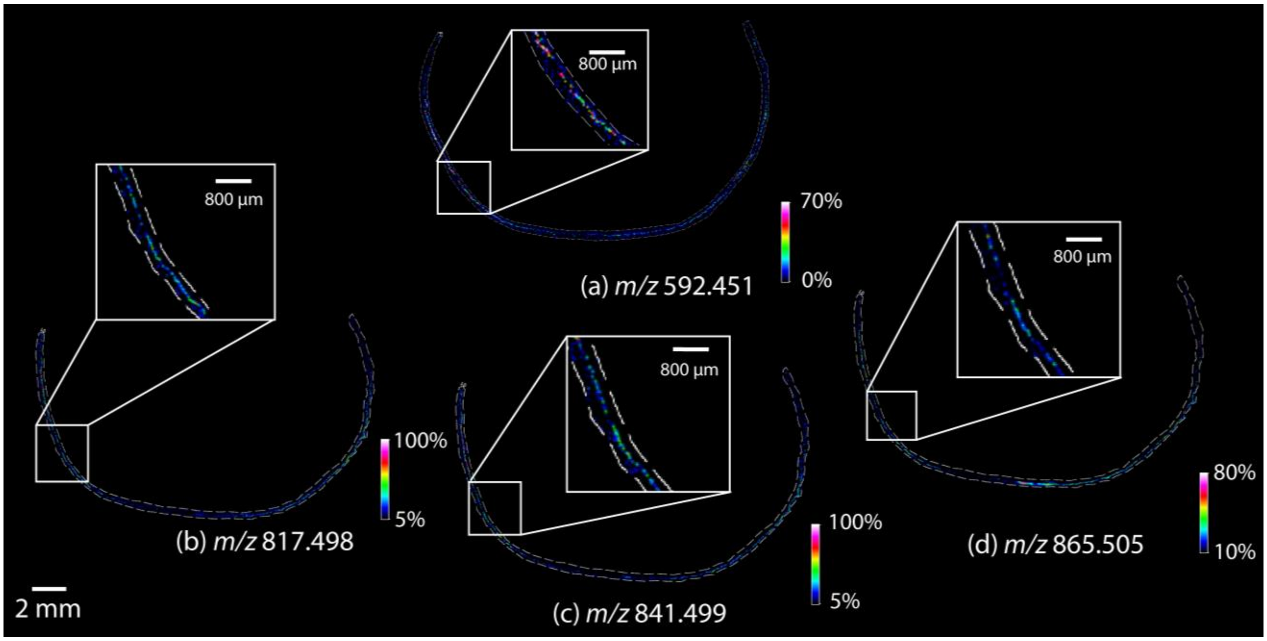


Supplementary figure 13. MALDI IMS analysis of 72 year old human retina tissue from sections adjacent to those in supplementary fig 11. (a) MALDI IMS data in positive ion mode displaying the relative abundance of *m/z* 592.451. MALDI IMS data in negative ion mode displaying the relative abundance of (b) *m/z* 817.498 (c) 841.499 and (d) *m/z* 865.505. Enlarged insets show zoomed in regions of RPE tissue from corresponding regions of the adjacent sections.
